# Possible association between androgenic alopecia and risk of prostate cancer and testicular germ cell tumor: a systematic review and meta-analysis

**DOI:** 10.1186/s12885-018-4194-z

**Published:** 2018-03-12

**Authors:** Weijun Liang, Liuying Song, Zheng Peng, Yan Zou, Shengming Dai

**Affiliations:** Department of Clinical Laboratory, the Fourth Affiliated Hospital of Guangxi Medical University, No.1 Liushi Road, Liuzhou, Guangxi Zhuang Autonomous Region 545005 People’s Republic of China

**Keywords:** Androgenic alopecia, Prostate cancer, Testicular germ cell tumor, Risk, Association, Meta-analysis

## Abstract

**Background:**

A number of studies have investigated the association between androgenic alopecia (AGA) and cancer risk, but they have yielded inconsistent results. Therefore, this study was conducted to explore this controversial subject.

**Methods:**

A literature database search was performed according to predefined criteria. An odds ratio (OR) or a hazard ratio (HR) with 95% confidence intervals (CIs) was retained to evaluate the relationship between the incidence of cancer or cancer-specific mortality and categories of AGA. Then a pooled OR or HR was derived.

**Results:**

The pooled results showed that no specific degree of baldness had an influence on the incidence of cancer or cancer-specific mortality. However, AGA, especially frontal baldness, with the incidence of testicular germ cell tumor (TGCT) (OR = 0.69; 95% CI = 0.58–0.83). A significant increase of risk was observed in relation to high grade prostate cancer (PC) (OR = 1.42; 95% CI 1.02–1.99) and vertex with/without frontal baldness was associated with PC risk.

**Conclusions:**

The study results supported the hypothesis that AGA is negatively associated with TGCT risk and suggested an overlapping pathophysiological mechanism between them, while the viewpoint that AGA can be used as a phenotypic marker for PC risk was poorly supported.

**Electronic supplementary material:**

The online version of this article (10.1186/s12885-018-4194-z) contains supplementary material, which is available to authorized users.

## Background

Androgenic alopecia (AGA) is characterized by nonscarring progressive reduction in the diameter, pigmentation, density, and length of hair from frontotemporal and/or vertex regions of the scalp in a distinctive pattern [1]. It is a type of androgen-dependent hair loss disorder that affects approximately 50% of men in their 50s, [2] and its prevalence and extent tends to increase with age [3]. A vital alteration to the hair cycle for AGA is that the duration of the anagen phase decreases stepwise while that of the telogen phase increases, [4] but the pathogenesis of this disorder, as yet, is not fully understood. However, it has been fairly well established that androgens, mainly dihydrotestosterone (DHT) as major regulators, have critical effects on human androgen-sensitive hair follicles and may inhibit follicles on certain areas of scalp in genetically susceptible individuals and therefore causing AGA [5]. Testosterone, the principal androgen circulating in males, is converted to DHT by 5-αreductase. It has been reported that 5α-reductase inhibitors, such as finasteride, have increased scalp hair [6]. In addition, it has also been determined that heritability plays a critical role in the miniaturization of hair follicles leading to a change in the hair cycle [7, 8].

A high prevalence of abdominal obesity, hypertension, and lower high-density lipoprotein was found in patients affected by AGA [9]. Moreover, numerous studies have shown that AGA is implicated in an increased risk of metabolic syndrome [10] and coronary heart disease [11]. AGA is not simply recognized as an abnormal benign symptom with psychological effects and cosmetic impacts, but it has been represented as a forbear for future potential chronic diseases and therefore deserves attention. In consideration of similar pathophysiological mechanisms in terms of age dependency, genetic predisposition, and hormone dependency [12, 13], researchers have hypothesized a link between AGA and cancer risk, especially hormone-related cancer. Since cancer remains a worldwide public health issue that threatens human life, early diagnosis such as tumor screening could be an effective way to reduce the incidence of cancer. Yet, there are still some obstacles that hinder the wide application of tumor screening, for example, the lack of pertinence and the substantial consumption of healthcare resources. In this sense, it is beneficial to specify the high-risk groups to be screened so that it can reduce the costs, and that is why AGA is now being considered as a vital sign.

For the past two decades, a considerable number of publications have studied the relationship between AGA and hormone-related cancer, but they haven’t yielded consistent results. Amoretti et al. [14] conducted a meta-analysis to reveal the relationship between AGA and prostate cancer in 2013, but there have been several published studies that involved more kinds of cancers. As a result, we conducted a systematic review and a comprehensive meta-analysis in order to further investigate the issue and identify potential sources of heterogeneity that might be confounders that have affected some existing conclusions.

## Methods

### Search strategy and selection criteria

We searched for all eligible publications that evaluated AGA and cancer risk in Embase and PubMed up to June 2016.The combined search strategy employed the terms *androgenic alopecia, alopecia, baldness, bald or balding* in combination with *cancer, tumor, or neoplasm* in combination with *risk, incidence*, or *mortality*. No language or country filters were imposed. The details of the searching terms were listed in (Additional file 1: Table S1). The selection criteria were as follows: (1) the studied participants were exposed to AGA, and there was no gender limitation; (2) the study evaluated the incidence or mortality of prostate cancer and testicular germ cell tumor; (3) the papers provided relative risks (RRs), including cancer-specific hazard ratios (HRs) or odds ratios (ORs) in combination with 95% confidence intervals (CIs), or provided related data could calculate RRs; and (4) the study design was unrestricted. If multiple publications reported overlapping data or the same data, the one with greater size or more information would be chosen. Reviews, comments, letters, notes, abstract and repeated literature case reports were all excluded.

### Data extraction and quality assessment

Two reviewers (L.S and M.D) independently retrieved the information from all eligible records. The methodological quality was assessed by two authors (W.L and L.S) using the Newcastle-Ottawa Scale (NOS) with 0–3 scores defined as low quality, 4–6 scores as moderate quality, and 7–9 scores as high quality. For each study, the participant and study characteristics, number of subjects, type of controls, study design, follow-up time, type of cancer, means of AGA assessment, method of case confirmation, cohort/control selection, and AGA categories were extracted and transformed into the specially designed forms. Disagreements or uncertainties were resolved by the reviewers’ re-verification of the data. If an agreement was not still reached, an additional adjudicator (S.D) was invited into the discussion.

### Data synthesis and statistical analysis

If available, multivariate-adjusted risk estimates were used for each study; otherwise, unadjusted RRs, which were calculated according to exposure distributions given in the papers, were utilized. When a study only provided risk estimates from the comparison of a subset, i.e. a specific category of hair pattern such as frontal/vertex baldness with the group of no baldness, the alternative estimates were synthesized as the summarized estimates for overall exposure. If risk estimates were presented as RRs or ORs, combined estimates were generated using the method proposed by Hamling et al. [15], and if they were presented as HRs, a fixed-effect model was conducted.

Considering potential interactions, studies were stratified by the different designs of the studies with the effects estimated by HRs for cohort studies or ORs for case-control studies. As the ORs for case-control studies approximate the RRs in cohort studies with low incidence and effects estimated generally approaching 1.0, the combination of ORs and RRs was permitted [16]. According to the results of inter-study heterogeneity appraisal using χ^2^-based Q statistics and I^2^ for statistical significance of heterogeneity, pooled ORs and HRs with 95% CI were calculated using a fixed-effect model (Mantel-Haenszel method) or random-effect model (DerSimonian-Laird method). A *P* value of Q statistics > 0.10 and I^2^ < 50% indicated little heterogeneity. Sensitivity analysis was performed to assess the influence of a single trial in the meta-analysis estimated by sequential omission of individual trials. Publication bias was assessed with a funnel plot and the Egger regression. An overall meta-analysis was carried out in all included studies, and then a specific type of cancer with over two articles was included as the subtype that was performed for further meta-analysis. Prespecified subgroup analyses were performed by, reference age (20, 30, 40, or 45) for baldness assessment, pattern of baldness (frontal, vertex, vertex with frontal, vertex with/without frontal, and frontal with vertex), amount of baldness [1st stage: I; 2nd stage: II, IIa; 3rd -4th stage: III, IIIa, III-vertex, IV, Iva; 5th–7th stage: V, Va, VI, VII which were measured by Hamilton-Norwood scale (see Additional file 2: Figure S1)], type of control, baldness assessment type (by self-reporting and by trained observers) and age of the case.

A two-sided *P* < 0.05 was considered statistically significant. The STATA version 12.0 (StataCorp LP, College Station, Texas, USA) was used for statistical analysis.

## Results

### Eligible studies

**Fig. 1:**
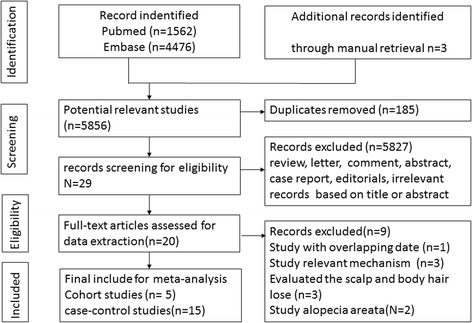
Flow diagram of included studies for this meta-analysis

The literature retrieval identified 1562 records in Pubmed and 4476 in Embase. Ultimately, 20 publications [17–35] were finally selected for the study; the flow diagram was presented in Fig. 1. The results of methodological quality assessment indicated that all included records were of high quality (More details were shown in Additional file 3: Table S2 and Table S3). All data generated or analysed during this study are included in this published article [and its Additional file 4: Excel S1].

### Baseline characteristics

In total, six cohort studies [17, 18, 20, 23, 24, 27] and 14 case-control studies [19, 21, 22, 25, 26, 28–36] were included in the present systematic review and meta-analysis. Among these, two cohort studies [17, 18, 37] were conducted on PC-specific mortality. In the case-control studies, ten studies addressed PC [21, 22, 26, 28, 30–32, 34–36], and four addressed TGCT [19, 25, 29, 33]. PC and TGCT were performed as the subtype for further meta-analysis. The baseline characteristics were shown in Table 1.

**Table 1:** Characteristics of included studies: androgenic alopecia and cancer risk

Study	Study location	Type of cancer	Case (number)	All subjects (number)	Follow-up time	Baldness Assessment	Case confirm	Baldness categories	Cohort
Cohort studies									
Zhou-3 2016	USA	PC	107	4316	21	trained observers	medical records	No pattern	no history of PC at baseline and other cause for blading and other cause for blading no history of PC at baseline and other cause for blading
Sarre 2016	Finland	PC	757	11,795	6.6	self-reported	database	9–11	no history of PC
Zhou-1 2015	USA	PC	2306	32,583	9.0	self-reported	medical records	1–2, 4	no history of any cancer
Zhou-2 2015	USA	PC	1138	39,070	2.8	self-reported	medical records	1–2, 6–8	no history or treatment of PC/CRC/LC
Muller 2012	Australia	PC	476	9448	11.4	self-reported	database	1–2, 4	Men> 45 years and no history of PC at baseline
Hawk 2000	USA	PC	214	4207	18.2	trained observers	medical records	No pattern	no history of PC and other cause for blading
Case-control studies				Control (number)	Exposure period				Control
Zeigler-Johnson 2013	USA	PC	219	318	1998–2010	self-reported	medical records	1–2, 4	Cancer-free
Thomas 2013	USA	PC	167	312	2007–2011	self-reported	histopathology	1–2, 4	PC -free
Yassa 2011	France	PC	388	281	2004–2006	self-reported	databases	1–3, 5	PC -free
Wright 2010	USA	PC	999	942	2002–2005	self-reported	databases	1–2, 4	PC -free
Cremers 2010	Netherlands	PC	938	2160	2003–2006	self-reported	medical records	1–3, 5	PC -free
Faydaci 2008	Turkey	PC	44	108	2005–2006	NR	histopathology	1–2, 4	BPH
Giles 2002	Australia	PC	1446	1390	1994–1997	trained observers	histopathology	1–3 5	PC -free
Demark-Wahnefried-1 2000	USA	PC	134	145	1993–1995	self-report	medical records	1–2, 4	Healthy or BPH or benign genitourinary disease
Hsieh 1999	USA	PC	320	246	1994–1997	self-report	histopathology	1–2, 4	Cancer free or BPH free with ENT disease
Demark-Wahnefried-2 1997	USA	PC	129	139	1993–1995	Trained observers	NR	1–2, 4	BPH
Moirano 2016	Italy	TGCTs	253	455	1997–2008	self-reported	histopathology	No pattern	No hormonal or infertility related neoplasm
Trabert 2011	USA	TGCTs	187	148	1990–1994	self-reported	databases	12	NR
Farzana 2002	USA	TGCTs	159	136	1990–1996	self-report	databases	No pattern	TGCTs-free
Petridou 1997	Greece	TGCTs	97	198	1993–1994	Trained observers	histopathology	12	healthy men

*BPH* prostatic hyperplasia, *ENT* Ear Nose Throat Depart, *UC* ulcerative colitis, *NR* not report, *LC* lung cancer, *CRC* colorectal cancer, *TGCTs* testicular germ cell tumor, *PC* prostate cancer

baldness categories: 1.no balding; 2. frontal balding; 3. vertex balding; 4. Vertex with/without frontal baldness5. frontal baldness with vertex baldness; 6. frontal with mild vertex baldness; 7. frontal with moderate vertex baldness; 8. frontal with severe vertex baldness benign; 9. no baldness; 10.frontal and/or vertex baldness; 11.almost or completely bald;12.1st-7th stage

### Systematic review and meta-analysis

The pooled results indicated that any kind of baldness had no influence on the PC-specific mortality in a random-effect model, with an HR = 1.07 (95% CI 0.43–2.64) and moderated heterogeneity (I^2^ = 69.2; *P* = 0.071). Twenty observational studies were involved in assessing the relationship between AGA and cancer incidence, including 6 cohort studies [17, 18, 20, 23, 24, 27] and 14 case-control studies [19, 21, 22, 25, 26, 28–36]. The cohort study of Hawk et al. [27] with the effects estimated by RRs was combined with case-control studies in the pooled analyses. A negative association was shown when all studies were pooled, with an HR = 0.99 (95% CI 0.91–1.09). Depending on the particular method of baldness assessment, the studies were separated into two subsets. The first subset included 10 studies [20, 22, 26, 27, 29, 31–35] that assessed baldness by self-reporting, and the second subset included 3 [21, 25, 28] studies where it was assessed by trained observers. No association was consistent in the two subsets. A summary of the results was presented in (Additional file 5: Table S4).

### Analysis of TGCT

**Fig. 2:**
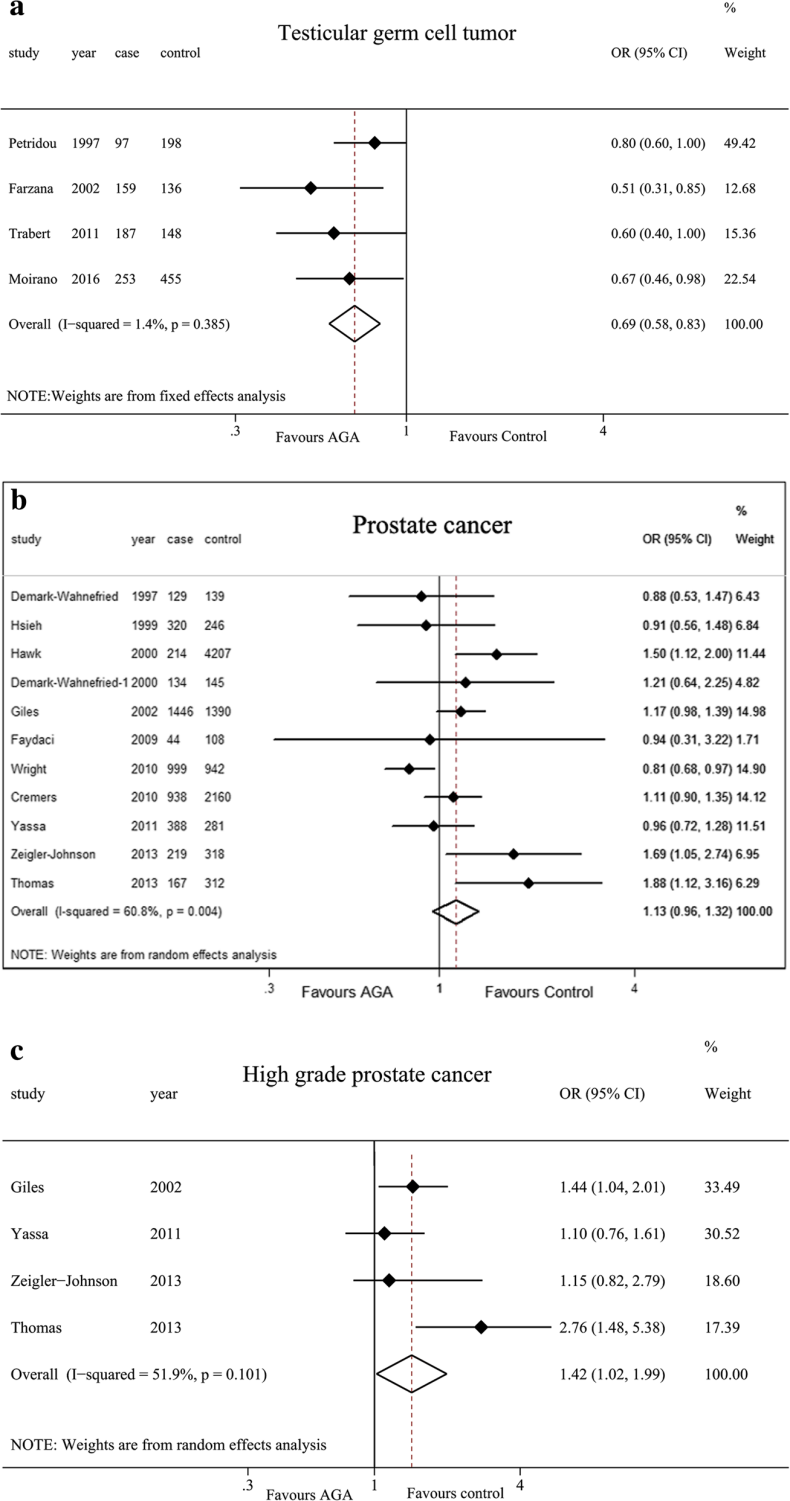
Forest plots of any AGA and the risk of PC and TGCT incidence for case-control studies: **a** for TGCT incidence; **b** for PC incidence; and **c** high grade PC incidence

Four case-control studies [19, 25, 29, 33] that assessed the influence of AGA on the incidence of TGCT showed a negative association (OR = 0.69; 95% CI 0.58–0.83; *P* < 0.001) with little evidence of heterogeneity (Fig. 2a). When the studies were stratified by histological subtypes, the results were consistent and more strongly evident in nonseminoma. When the studies were stratified by different degree of baldness on the basis of the Hamilton-Norwood scale, hair loss at 2nd stage was negatively correlated with TGCT risk compared to that at 1ST stage (OR = 0.46; 95% CI 0.30–0.72; *P* = 0.001). A summary of the results was presented in Table 2.

**Table 2:** Meta-analysis results of association between AGA and incidence of testicular germ cell tumor

Study characteristics	Number of studies	OR(95% CI)	*P* value	Effect model	Heterogeneity	
					I^2^ (%)	*P* value
Overall	4	0.69 (0.58–0.83)	< 0.001	fixed	1.4	0.385
Seminoma	4	0.71(0.55–0.93)	0.011	fixed	< 0.1	0.545
Nonseminoma	4	0.57(0.44–0.74)	< 0.001	fixed	43.2	0.153
Baldness assessment type						
self-reported	3	0.61(0.47–0.78)	< 0.001	fixed	< 0.1	0.697
Different amount of baldness						
2nd vs. 1st stage	2	0.46(0.30–0.72)	0.001	fixed	< 0.1	0.639
3rd -4th vs. 1st stage	2	0.67(0.40–1.13)	0.135	fixed	< 0.1	0.794
5th -7th vs. 1st stage	2	0.46(0.20–1.05)	0.065	fixed	< 0.1	0.797

### Case-control studies & analysis of PC

The pooled OR for the 11 studies [21, 22, 26–28, 30–32, 34–36] that assessed the association between any AGA and the risk of PC was 1.13 (0.96–1.32) (Fig. 2b). However, when studies on PC stratified by different grades/stages [22, 28, 34, 35] were combined, a significantly increased risk was observed in high grade PC (OR = 1.42; 95% CI 1.02–1.99; *P* = 0.038) (Fig. 2c). The effect of size demonstrated a moderate statistical heterogeneity among studies of PC. When studies of PC were stratified by grades, no evidence of heterogeneity was observed. All the meta-analysis results of association between AGA and incidence of PC are listed in Table 3.

**Table 3:** Meta-analysis results of association between AGA and incidence of prostate cancer

Study characteristics	Number of studies	OR(95% CI)	*P* value	Effect model	Heterogeneity	
					I^2^ (%)	*P* value
Case-control studies	11	1.13(0.96–1.32)	0.150	random	60.8	0.004
high grade^a^	3	1.46(0.89–2.51)	0.172	random	67.3	0.047
high grade^b^	4	1.42 (1.02–1.99)	0.038	random	51.9	0.101
high stage	2	1.29(0.61–2.72)	0.503	random	59.4	0.117
Baldness assessment type						
self-reported	8	1.15(0.94–1.41)	0.178	random	66.5	0.002
trained observers	2	1.13(0.93–1.36)	0.215	fixed	6.7	0.300
Different patterns of baldness						
Frontal vs. No	10	1.03 (0.86–1.23)	0.786	random	48.5	0.042
Vertex with/without Frontal vs. no baldness	6	1.29(1.03–1.61)	0.029	fixed	9.8	0.353
Vertex without Frontal vs. no baldness	3	1.23(0.95–1.60)	0.124	random	58.1	0.092
Frontal with Vertex vs. no baldness	4	1.01(0.89–1.15)	0.899	fixed	< 0.1	0.524
Different reference age						
20	2	1.28(0.59–2.75)	0.533	random	81.4	0.020
30	5	1.25(0.83–1.88)	0.289	random	79.5	0.001
40	4	1.00(0.78–1.29)	0.988	random	52.4	0.098
Different age of case						
< 60	3	1.11(0.92–1.35)	0.264	fixed	27.8	0.251
≥ 60	3	1.03(0.68–1.55)	0.902	random	71.2	0.031
Different type of control						
PC-free	5	1.07(0.87–1.31)	0.516	random	73.3	0.005
BPH	2	1.20(0.72–2.01)	0.490	random	72.0	0.002
Cohort studies	4	0.99(0.94–1.05)	0.714	Fixed	< 0.1	0.521
Subtypes of cancer	5	1.02(0.93–1.13)	0.656	random	59.5	0.043
Frontal vs. no baldness	2	1.01(0.93–1.10)	0.756	fixed	< 0.1	0.831
aggressive	2	1.01(0.94–1.14)	0.812	fixed	< 0.1	0.876
nonaggressive	2	1.07(0.96–1.20)	0.194	fixed	< 0.1	0.701
Frontal with Vertex vs. no baldness	2	1.05(0.96–1.14)	0.289	fixed	< 0.1	0.914
aggressive	2	1.04(0.93–1.17)	0.490	fixed	< 0.1	0.641
nonaggressive	2	1.01(0.89–1.14)	0.894	fixed	< 0.1	0.483
reference age = 45	2	1.01(0.94–1.071)	0.858	fixed	< 0.1	0.780

*BPH* prostatic hyperplasia

high grade^a^: Gleason score 7–10; high grade^b^: Gleason score 8–10 and Gleason score 7–10; high stage: T-stage 3–4; aggressive prostate cancer: Gleason score 7–10 or regional/distant metastases (SEER summary stage) or fatal prostate cancer

#### Different patterns of baldness

Based on the Hamilton-Norwood Scale, the 8 study subsets [21, 22, 26, 27, 30, 32, 33, 35] and the 4 study subsets [25, 28, 31, 34] were categorized for male pattern baldness, among which 3 variables (none or little baldness, frontal baldness and vertex with/without frontal baldness) were used for the first subset and 4 variables (none or little baldness, frontal, vertex without frontal baldness, and frontal with vertex) for the second subset. Vertex with/without frontal baldness was associated with PC (OR = 1.29; 95% Cl 1.03–1.61; *P* = 0.029).

#### Different reference age

A total of two studies evaluated AGA at reference age 20 [31, 34], five at age 30 [22, 32, 34–36], and four at age 40 [31, 34–36], respectively. No association was established for participants with AGA at ages 20, 30, and 40, and these analyses yielded moderate heterogeneity.

#### Different age of case

Three studies [22, 28, 32] calculated the age-stratified association. AGA was not associated with the risk of cancer incidence in either younger men (< 60 years of age) or older men (≥ 60 years of age).

#### Different types of controls

As controls, five studies [28, 31, 32, 34, 35] selected PC-free participants, two [21, 30] selected prostatic hyperplasia (BPH) participants, and the others selected cancer-free or benign disease participants. No significant relationship was found between AGA and PC risk when the study was stratified by the different types of controls.

### Cohort studies & analysis of PC

When four cohort studies [17, 18, 23, 24] were combined, the pooled results revealed that AGA had no relationship with prostate cancer in a random-effect model, with an HR = 0.99(0.94–1.05) and moderated heterogeneity (I^2^ = < 0.1%). In other subgroup analysis, the results were consistent.

### Sensitivity analysis and publication bias

**Fig. 3:**
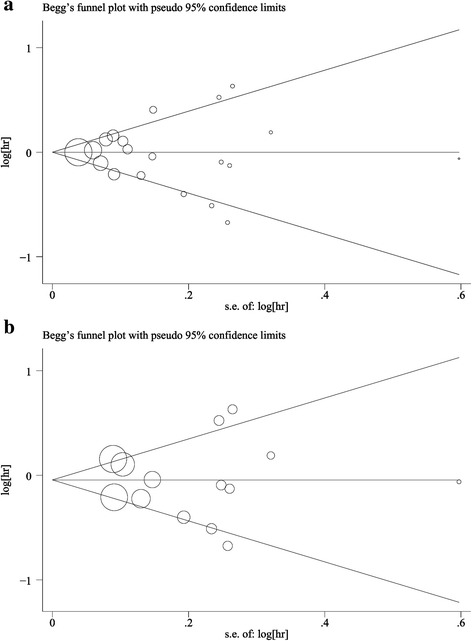
Begg’s funnel plots for publication bias of the relative risk of cancer incidence: **a** for all included studies; **b** for case-control studies; and **c** for cohorts

Sensitivity analysis confirmed that no individual study influenced the overall results (data not shown). There was no evidence of publication bias in this meta-analysis indicated by the Begg’s funnel plot and Egger’s tests (Fig. 3).

## Discussion

This systematic review and meta-analysis involved a total of 10,935 cases from 21 observational studies. Amoretti et al. [14] conducted a meta-analysis to examine the association between AGA and the incidence of PC, but more studies have been published to indicate its relation to other kinds of cancer. Besides, this meta-analysis also evaluated the relationship between AGA and the mortality of cancer. Moreover, several subgroup analyses were conducted to evaluate whether the association varied by subtype of PC or TGCT, reference age for baldness assessment, pattern of baldness, type of control, and age of the case, and to minimize potential confounding biases.

Cases of AGA demonstrated a 31% decrease in the incidence of TGCT compared to those with no baldness. The sample size was not sufficient as there were only four studies included in the subset but little heterogeneity was shown. AGA may be regarded as a surrogate of cumulative androgen status which was considered as the result of its components such as exogenous hormones, endogenous hormonal levels, hormonal metabolism, or individual sensitivity to hormones. TGCT is significantly different from PC at the onset age. High risk for TGCT often occurs at ages 20–45 while it occurs at later ages for PC. It has been reported that circulating testosterone, DHT, and E2 declined gradually during male aging [38]. Thus, age differences both in the incidence of different kinds of cancer and the androgen status account for a complex interplay of these four components. In addition, androgen receptor and androgen metabolic pathway genetic variation studies showed that Ser312Asn polymorphism of the luteinizing hormone receptor was linked to an increased relative risk of PC [39] and a decreased relative risk of TGCT [40]. This suggested that androgen expression and metabolism might have tissue-specific effects. Moreover, testosterone and DHT appeared to be tissue-specific as well [41]; DHT played a vital role primarily in prostate and scalp, while testosterone appeared to function in spermatogenesis, muscle, and bone. These findings suggested that a complex interaction may partly explain the reason why AGA, as a surrogate of high androgen, has been implicated in different cancer but has shown conflicting results. AGA might reflect androgen status more directly and be less affected by other factors, thus being inversely related to tumorigenesis in TGCT. In histologic specific analyses on TGCT, the pooled results indicated that AGA exposure was significantly inversely related to the risks of developing both seminoma and nonseminoma. This relationship was more predominant among nonseminoma with less heterogeneity. Given that nonseminoma is often diagnosed at ages 25–29 on average, which is 10 years earlier than seminoma [42], the risk of TGCT decreased due to hormonal related factors during this time, and they may be more relevant to nonseminoma. Also, it was possible that these differences were caused by an insufficient sample size and inadequate statistical power for stratified analysis.

For 11 of the included studies, the association of AGA with PC was not evident in the overall population, which was consistent with the previous study [42]. Some authors speculated that the earlier onset of AGA, compared with its later onset, is a risk factor for developing PC. However, all the pooled results at different AGA time points (20, 30, 40, and 45) were negative. Yet, the interference of another potential source of bias, such as inadequate sample size, recall and selection bias, and study design, could not be ruled out. But, the present results could not support this interference. Also, the pooled results for baldness assessment type, age of case, and control selection were thought to be major factors. Coincident with the overall results, no subset analyses could establish a link between AGA and the incidence of PC. Thus, it is plausible that the overall results are robust. In addition, moderate inter-study heterogeneity was found to be consistent in case-control studies which suggested that there might be some other confounders or bias that accounted for inter-study heterogeneity. Several studies [22, 28, 34, 35] categorized the cases of PC into two grades according to Gleason scores, but the cut-off scores were not congruent. A negative result was achieved when included studies only focused on high grade PC with Gleason scores of 7–10. It was interesting to note that the result turned out to be positive when a study on high grade PC with Gleason scores of 8–10 was included. However, the pooled results showed AGA was not linked to aggressive PC (defined as Gleason scores of 7–10, regional/distant metastases, or fatal prostate cancer) without a difference in any baldness pattern in the cohort studies. The Gleason score was regarded as a good indicator of PC aggressiveness. Gleason 7 was divided into Gleason 4 + 3 and 3 + 4. Pathologically advanced PC and poor prognosis were more common in the first of the two. In our present meta-analysis, however, an agreement on the exact grade of PC with Gleason score 7 was not reached. A new Gleason grading system was proposed wherein Gleason scores ≤6 were lumped into prognostic grade group I, the score of 3 + 4 = 7 into group II, the score of 4 + 3 = 7 into group III, the score of 4 + 4 = 8 into group IV, and scores of 9–10 into group V [43, 44]. Thus, whether the less aggressive Gleason 7 cases account for the negative results or not needed to be confirmed and a new Gleason grading system should be applied in further studies.

When it came to different categories of baldness, the results showed that the vertex pattern with/without frontal baldness was related to PC risk, but the frontal pattern was not. On the other side, baldness at 2nd stage which amounted to frontal baldness was related to TGCT risk, while the other categories were not. As a result, a dose-response relationship could not be obtained. If these relationships were real, they may be possibly explained by non-linear correlations between the degree of AGA and the circulating androgen status. It was difficult to explain how the results turned to be negative in the PC, when vertex without frontal baldness and vertex with frontal baldness were regarded as independent patterns in the pooled studies. Also, no specific pattern of AGA showed any link to PC risk in the cohort studies. The pathophysiological difference among the patterns of AGA was yet unknown, so a reasonable explanation could not be provided. In addition, the reference age of AGA was different in the included studies; however, Muller et al. [20] found that vertex AGA at age 40 was not associated with the risk of PC; at age 55, the vertex AGA group had a higher hazard of prostate cancer; at ages 60–70, the HR was not discernible from 1; and at age 75, the hazard of prostate cancer was lower. This indicated that the association between AGA and PC was by nature age-variant and could not be sufficiently described by a single, age-invariant estimate of relative risk. Thus, the association might be masked by inconsistent AGA assessment age. Moreover, finasteride is a type II 5-αreductase inhibitor and is commonly used to treat AGA, [37] which could theoretically decrease the incidence of PC [45]. Another important point was that numerous epidemiologic studies have shown that AGA was associated with cardiovascular disease [46]. However, most of these studies lacked information on the connection of comorbidity and the use of finasteride. Therefore, this connection was potentially a major source of bias that could influence the final results, and the positive results should be interpreted with caution.

In this meta-analysis, only two of the included studies examined the association of AGA and PC-specific mortality, which showed that inner-study heterogeneity was moderate. Hence, more research work was needed to confirm these findings.

Nevertheless, several limitations of this study must be acknowledged. First, despite several subgroup analyses to be performed, significant heterogeneity was generally observed. Given the differences of the studies in race, age, participants’ lifestyle, information collection method, sample size, duration of follow-up and so on, heterogeneity was not avoidable. Second, the number of stratified analyses was so limited that might result in invalid statistical analyses in those groups. Third, although most of the studies used multivariate statistical models to calculate the estimated RRs, the number and content of the adjusted confounders varied in each trial, which might lead to imprecision in the results. But, the most multivariable adjusted-effect estimates were chosen for analysis to minimize the confounding biases. Besides, several sources of bias, such as inherent limitations, unmeasured confounding, and the typical bias of observational studies, could have affected the observed results. Therefore, well-designed and more comprehensive studies are still needed to further evaluate the relationship between AGA and the risk of cancers.

## Conclusions

The results support the hypothesis that AGA is associated with a reduction of TGCT incidence by altering testicular development and they also suggest that there is a common pathogenic pathway. For PC, positive results are only observed in vertex patterns of AGA and high grade PC, while the viewpoint that AGA can be used as a phenotypic marker for PC risk is poorly supported. In this sense, future studies should be conducted to confirm the conclusions, as well as to evaluate the potential value of this association, which may offer a reference for establishment of predictive models.

## Additional files

Additional file 1: **Table S1.** Review methodology for meta-analysis. (DOC 29 kb)

Additional file 2: **Figure S1.** Male balding patterns base on the Hamilton-Norwood scale. (TIFF 356 kb)

Additional file 3: **Table S2.** NOS scores of cohort studies. **Table S3.** NOS scores of case control studies. (DOC 86 kb)

Additional file 4: **Excel S1.** Extracted raw data in the meta-analysis. (XLSX 16 kb)

Additional file 5: **Table S4.** Meta-analysis results of association between androgenic alopecia and incidence of cancer. (DOCX 17 kb)

## Abbreviations

AGA: Androgenic alopecia
BPH: Prostatic hyperplasia
CIs: Confidence intervals
DHT: Dihydrotestosterone
HRs: Hazard ratios
NOS: Newcastle-Ottawa Scale
ORs: Odds ratios
PC: Prostate cancer
RRs: Relative risks
TGCT: Testicular germ cell tumor

## References

[1] Piraccini B, Alessandrini A (2014). Androgenetic alopecia. G Ital Dermatol Venereol.

[2] Kaliyadan F, Nambiar A, Vijayaraghavan S (2013). Androgenetic alopecia: an update. Indian J Dermatol, Venereol Leprol.

[3] Hamilton JB (1951). Patterned loss of hair in man: types and incidence. Ann N Y Acad Sci.

[4] Randall VA (2008). Androgens and hair growth. Dermatol Ther.

[5] Kaufman KD (1996). Androgen metabolism as it affects hair growth in androgenetic alopecia. Dermatol Clin.

[6] Blumeyer A, Tosti A, Messenger A, Reygagne P, Del Marmol V, Spuls PI, Trakatelli M, Finner A, Kiesewetter F, Trüeb R (2011). Evidence-based (S3) guideline for the treatment of androgenetic alopecia in women and in men. J Dtsch Dermatol Ges.

[7] Nyholt DR, Gillespie NA, Heath AC, Martin NG (2003). Genetic basis of male pattern baldness. J Investig Dermatol.

[8] Li R, Brockschmidt FF, Kiefer AK, Stefansson H, Nyholt DR, Song K, Vermeulen SH, Kanoni S, Glass D, Medland SE (2012). Six novel susceptibility loci for early-onset androgenetic alopecia and their unexpected association with common diseases. PLoS Genet.

[9] Gopinath H, Upadya GM (2016). Metabolic syndrome in androgenic alopecia. Indian J Dermatol Venereol Leprol.

[10] Wu D, Wu L, Yang Z (2014). Association between androgenetic alopecia and metabolic syndrome: a meta-analysis. Zhejiang Da Xue Xue Bao Yi Xue Ban.

[11] Trieu N, Eslick GD (2014). Alopecia and its association with coronary heart disease and cardiovascular risk factors: a meta-analysis. Int J Cardiol.

[12] Henderson BE, Ross RK, Pike MC, Casagrande JT (1982). Endogenous hormones as a major factor in human cancer. Cancer Res.

[13] Folkerd EJ, Dowsett M (2010). Influence of sex hormones on cancer progression. J Clin Oncol.

[14] Amoretti A, Laydner H, Bergfeld W (2013). Androgenetic alopecia and risk of prostate cancer: a systematic review and meta-analysis. J Am Acad Dermatol.

[15] Hamling J, Lee P, Weitkunat R, Ambühl M (2008). Facilitating meta-analyses by deriving relative effect and precision estimates for alternative comparisons from a set of estimates presented by exposure level or disease category. Stat Med.

[16] Zhang J, Yu KF (1998). What’s the relative risk? A method of correcting the odds ratio in cohort studies of common outcomes. JAMA.

[17] Zhou CK, Levine PH, Cleary SD, Hoffman HJ, Graubard BI, Cook MB (2016). Male pattern baldness in relation to prostate Cancer-specific mortality: a prospective analysis in the NHANES I epidemiologic follow-up study. Am J Epidemiol.

[18] Sarre S, Määttänen L, Tammela TLJ, Auvinen A, Murtola TJ. Postscreening follow-up of the Finnish prostate Cancer screening trial on putative prostate cancer risk factors: vitamin and mineral use, male pattern baldness, pubertal development and non-steroidal anti-inflammatory drug use. Scand J Urol. 2016;50(4):267–73. 10.3109/21681805.2016.1145734 26927237

[19] Moirano G, Zugna D, Grasso C, Lista P, Ciuffreda L, Segnan N, Merletti F, Richiardi L (2016). Baldness and testicular cancer: the EPSAM case-control study. Andrology.

[20] Muller DC, Giles GG, Sinclair R, Hopper JL, English DR, Severi G (2013). Age-dependent associations between androgenetic alopecia and prostate cancer risk. Cancer Epidemiol Biomark Prev.

[21] Demark-Wahnefried W, Lesko SM, Conaway MR, Robertson CN, Clark RV, Lobaugh B, Mathias BJ, Strigo TS, Paulson DF (1997). Serum androgens: associations with prostate cancer risk and hair patterning. J Androl.

[22] Zeigler-Johnson C, Morales KH, Spangler E, Chang BL, Rebbeck TR (2013). Relationship of early-onset baldness to prostate cancer in african-american men. Cancer Epidemiol Biomark Prev.

[23] Zhou CK, Littman AJ, Levine PH, Hoffman HJ, Cleary SD, White E, Cook MB (2015). Male pattern baldness in relation to prostate cancer risks: an analysis in the VITamins and lifestyle (VITAL) cohort study. Prostate.

[24] Zhou CK, Pfeiffer RM, Cleary SD, Hoffman HJ, Levine PH, Chu LW, Hsing AW, Cook MB (2015). Relationship between male pattern baldness and the risk of aggressive prostate cancer: an analysis of the prostate, lung, colorectal, and ovarian Cancer screening trial. J Clin Oncol.

[25] Petridou E, Roukas KI, Dessypris N, Aravantinos G, Bafaloukos D, Efraimidis A, Papacharalambous A, Pektasidis D, Rigatos G, Trichopoulos D (1997). Baldness and other correlates of sex hormones in relation to testicular cancer. Int J Cancer.

[26] Hsieh CC, Thanos A, Mitropoulos D, Deliveliotis C, Mantzoros CS, Trichopoulos D (1999). Risk factors for prostate cancer: a case-control study in Greece. Int J Cancer.

[27] Hawk E, Breslow RA, Graubard BI (2000). Male pattern baldness and clinical prostate cancer in the epidemiologic follow-up of the first National Health and nutrition examination survey. Cancer Epidemiol Biomark Prev.

[28] Giles GG, Severi G, Sinclair R, English DR, McCredie MR, Johnson W, Boyle P, Hopper JL (2002). Androgenetic alopecia and prostate cancer: findings from an Australian case-control study. Cancer Epidemio Biomarkers Prev.

[29] Walcott FL, Hauptmann M, Duphorne CM, Pillow PC, Strom SS, Sigurdson AJ (2002). A case-control study of dietary phytoestrogens and testicular cancer risk. Nutr Cancer.

[30] Faydaci G, Bilal E, Necmettin P, Fatih T, Asuman O, Ugur K (2008). Baldness, benign prostate hyperplasia, prostate cancer and androgen levels. Aging Male.

[31] Cremers RG, Aben KK, Vermeulen SH, Den Heijer M, Van Oort IM, Kiemeney LA (2010). Androgenic alopecia is not useful as an indicator of men at high risk of prostate cancer. Eur J Cancer.

[32] Wright JL, Page ST, Lin DW, Stanford JL (2010). Male pattern baldness and prostate cancer risk in a population-based case-control study. Cancer Epidemiol.

[33] Trabert B, Sigurdson AJ, Sweeney AM, Amato RJ, Strom SS, McGlynn KA (2011). Baldness, acne and testicular germ cell tumours. Int J Androl.

[34] Yassa M, Saliou M, de Rycke Y, Hemery C, Henni M, Bachaud JM, Thiounn N, Cosset JM, Giraud P (2011). Male pattern baldness and the risk of prostate cancer. Ann Oncol.

[35] Thomas JA, Antonelli JA, Banez LL, Hoyo C, Grant D, Demark-Wahnefried W, Platz EA, Gerber L, Shuler K, Eyoh E (2013). Androgenetic alopecia at various ages and prostate cancer risk in an equal-access multiethnic case-control series of veterans. Cancer Causes Control.

[36] Demark-Wahnefried W, Schildkraut JM, Thompson D, Lesko SM, McIntyre L, Schwingl P, Paulson DF, Robertson CN, Anderson EE, Walther PJ (2000). Early onset baldness and prostate cancer risk. Cancer Epidemiol Biomarkers Prev.

[37] Mella JM, Perret MC, Manzotti M, Catalano HN, Guyatt G (2010). Efficacy and safety of finasteride therapy for androgenetic alopecia: a systematic review. Arch Dermatol.

[38] Handelsman DJ, Yeap B, Flicker L, Martin S, Wittert GA, Ly LP (2015). Age-specific population centiles for androgen status in men. Eur J Endocrinol.

[39] Ingles SA, Liu SV, Pinski J (2013). LHRH and LHR genotypes and prostate cancer incidence and survival. Int J Mol Epidemiol Genet.

[40] Kristiansen W, Aschim E, Andersen J, Witczak O, Fosså S, Haugen T (2012). Variations in testosterone pathway genes and susceptibility to testicular cancer in Norwegian men. Int J Androl.

[41] Khera M, Crawford D, Morales A, Salonia A, Morgentaler A (2014). A new era of testosterone and prostate cancer: from physiology to clinical implications. Eur Urol.

[42] Gatta G, Trama A. Epidemiology of testicular Cancer. In: Colecchia M, editor. Pathology of testicular and penile neoplasms. New York City: Springer; 2016. p. 3–18.

[43] Chen N, Zhou Q (2016). The evolving Gleason grading system. Chinese journal of cancer research = Chung-kuo yen cheng yen chiu.

[44] Epstein JI, Egevad L, Amin MB, Delahunt B, Srigley JR, Humphrey PA (2016). The 2014 International Society of Urological Pathology (ISUP) consensus conference on Gleason grading of prostatic carcinoma: definition of grading patterns and proposal for a new grading system. Am J Surg Pathol.

[45] Vickers AJ, Savage CJ, Lilja H (2010). Finasteride to prevent prostate cancer: should all men or only a high-risk subgroup be treated?. J Clin Oncol.

[46] Yamada T, Hara K, Umematsu H, Kadowaki T (2013). Male pattern baldness and its association with coronary heart disease: a meta-analysis. BMJ Open.

