# Supplementary material for: Possible association between androgenic alopecia and risk of prostate cancer and testicular germ cell tumor: a systematic review and meta-analysis
Source: BMC Cancer. 2018 Mar 12;18:279. doi: 10.1186/s12885-018-4194-z (PMC5848631; doi:10.1186/s12885-018-4194-z)
Supplement: Supplementary file 1 — Table S1. Review methodology for meta-analysis. (DOC 29 kb) [file 12885_2018_4194_MOESM1_ESM.doc]

Table S1 Review methodology for meta-analysis

| **Electronic databases** | Embase and PubMed |
| --- | --- |
| **Search terms #1** | androgenic alopecia OR alopecia OR baldness OR bald OR balding OR AGA |
| **Search terms #2** | cancer OR tumor OR neoplasm |
| **Search terms #3** | Risk OR incidence OR mortality |
| **Final Search terms** | Search terms #1 AND Search terms #2 AND Search terms #3 |
