# Supplementary figures and images for: Possible association between androgenic alopecia and risk of prostate cancer and testicular germ cell tumor: a systematic review and meta-analysis
Source: BMC Cancer. 2018 Mar 12;18:279. doi: 10.1186/s12885-018-4194-z (PMC5848631; doi:10.1186/s12885-018-4194-z)

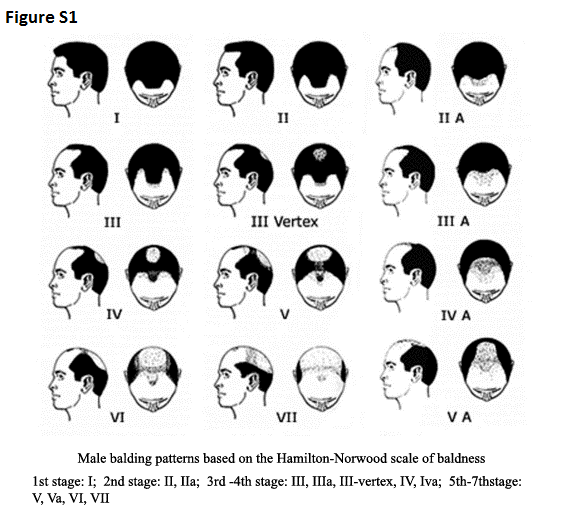

Supplement: Supplementary file 2 — Figure S1. Male balding patterns base on the Hamilton-Norwood scale. (TIFF 356 kb) [file 12885_2018_4194_MOESM2_ESM.tif]
