# Supplementary material for: Possible association between androgenic alopecia and risk of prostate cancer and testicular germ cell tumor: a systematic review and meta-analysis
Source: BMC Cancer. 2018 Mar 12;18:279. doi: 10.1186/s12885-018-4194-z (PMC5848631; doi:10.1186/s12885-018-4194-z)
Supplement: Supplementary file 3 — Table S2. NOS scores of cohort studies. Table S3. NOS scores of case control studies. (DOC 86 kb) [file 12885_2018_4194_MOESM3_ESM.doc]

Table S2 NOS scores of cohort studies

| **NOS scale** |  | Zhou-1  2016 | Sarre  2016 | Zhou-2  2015 | Zhou-3  2014 | Muller  2012 | Hawk  2000 |
| --- | --- | --- | --- | --- | --- | --- | --- |
| **Selection (maximum 4)** |  |  |  |  |  |  |  |
| 1.Representativeness of the exposed cohort |  | 1 | 1 | 1 | 1 | 1 | 1 |
| 2.Selection of the non exposed cohort |  | 1 | 1 | 1 | 1 | 1 | 1 |
| 3.Ascertainment of exposure |  | 1 | 1 | 1 | 1 | 1 | 1 |
| 4.Demonstration that outcome of interest was not present at start of study |  | 0 | 1 | 1 | 1 | 1 | 0 |
|  |  |  |  |  |  |  |  |
| **Comparability (maximum2)** |  |  |  |  |  |  |  |
| 1.Comparability of cohorts on the basis of the  design or analysis |  | 1 | 1 | 1 | 2 | 1 | 1 |
|  |  |  |  |  |  |  |  |
| **Outcome (maximum 3)** |  |  |  |  |  |  |  |
| 1.Assessment of outcome |  | 1 | 1 | 1 | 1 | 1 | 1 |
| 2.Was follow-up long enough for outcomes to occur |  | 1 | 1 | 1 | 1 | 1 | 1 |
| 3.Adequacy of follow up of cohorts |  | 1 | 1 | 1 | 1 | 1 | 1 |
|  |  |  |  |  |  |  |  |
| **Total (maximum 9)** |  | 7 | 8 | 8 | 9 | 8 | 7 |

Table S3 NOS scores of case control studies

| **NOS scale** | Moirano  2016 | Thomas  2013 | Zeigler-Johnson  2013 | Yassa  2011 | Trabert  2011 | Wright  2010 | Cremers  2010 | Faydaci  2009 |
| --- | --- | --- | --- | --- | --- | --- | --- | --- |
| **Selection (maximum 4)** |  |  |  |  |  |  |  |  |
| Case definition adequate | 1 | 1 | 1 | 1 | 1 | 1 | 1 | 1 |
| Representativeness of the case | 1 | 1 | 1 | 1 | 1 | 1 | 1 | 1 |
| Selection of control | 1 | 1 | 1 | 1 | 1 | 1 | 1 | 1 |
| Definition of control | 1 | 0 | 1 | 1 | 1 | 1 | 1 | 0 |
|  |  |  |  |  |  |  |  |  |
| **Comparability (maximum2)** |  |  |  |  |  |  |  |  |
| Comparability of cases and controls on the basis of the design or analysis | 1 | 1 | 1 | 1 | 1 | 1 | 1 | 1 |
|  |  |  |  |  |  |  |  |
|  |  |  |  |  |  |  |  |  |
| **Exposure (maximum3)** |  |  |  |  |  |  |  |  |
| Ascertainment of exposure | 1 | 1 | 1 | 1 | 1 | 1 | 1 | 1 |
| Same method cases and controls | 1 | 1 | 1 | 1 | 1 | 1 | 1 | 1 |
| Non-response rate | 1 | 1 | 0 | 1 | 0 | 0 | 1 | 0 |
|  |  |  |  |  |  |  |  |  |
| **Total (maximum 9)** | 8 | 7 | 7 | 8 | 7 | 7 | 8 | 6 |
| **NOS scale** | Giles  2002 | Farzana  2002 | Demark-Wahnefried-1  2000 | | Hsieh  1999 | Petridou  1997 | Demark-Wahnefried-2  1997 | |
| **Selection (maximum 4)** |  |  |  | |  |  |  | |
| Case definition adequate | 1 | 1 | 1 | | 1 | 1 | 1 | |
| Representativeness of the case | 1 | 1 | 1 | | 1 | 1 | 1 | |
| Selection of control | 1 | 1 | 1 | | 1 | 1 | 1 | |
| Definition of control | 1 | 1 | 1 | | 1 | 0 | 1 | |
| **Comparability (maximum2)** |  |  |  | |  |  |  | |
| Comparability of cases and controls on the basis of the design or analysis | 1 | 1 | 1 | | 1 | 1 | 1 | |
|  |  |  | |  |  |  | |
| **Exposure (maximum3)** |  |  |  | |  |  |  | |
| Ascertainment of exposure | 1 | 1 | 1 | | 1 | 1 | 1 | |
| Same method cases and controls | 1 | 1 | 1 | | 1 | 1 | 1 | |
| Non-response rate | 0 | 0 | 0 | | 0 | 0 | 1 | |
|  |  |  |  | |  |  |  | |
| **Total (maximum 9)** | 7 | 7 | 7 | | 7 | 6 | 8 | |
