# Supplementary material for: Possible association between androgenic alopecia and risk of prostate cancer and testicular germ cell tumor: a systematic review and meta-analysis
Source: BMC Cancer. 2018 Mar 12;18:279. doi: 10.1186/s12885-018-4194-z (PMC5848631; doi:10.1186/s12885-018-4194-z)
Supplement: Supplementary file 5 — Table S4. Meta-analysis results of association between androgenic alopecia and incidence of cancer. (DOCX 17 kb) [file 12885_2018_4194_MOESM5_ESM.docx]

**Table S4 Meta-analysis results of association between androgenic alopecia and incidence of cancer**

| Study | Number | OR, 95% CI | *P* value | Effect model | Heterogeneity | |
| --- | --- | --- | --- | --- | --- | --- |
| characteristics | of studies |  |  |  | I^2^ % | *P* value |
| **All studies** |  |  |  |  |  |  |
| **overall** | 20 | 0.99, 0.91-1.09 | 0.897 | random | 63.3 | <0.001 |
| ***baldness*** ***assessment way*** |  |  |  |  |  |  |
| self-reported | 14 | 1.00, 0.89-1.12 | 0.945 | random | 68.2 | <0.001 |
| trained observers | 4 | 0.95, 0.79-1.14 | 0.567 | random | 61.6 | 0.050 |
|  |  |  |  |  |  |  |
| **Case-control studies** |  |  |  |  |  |  |
| **overall** | 15 | 1.05(0.96-1.14) | 0.314 | random | 53.7 | 0.007 |
| high grade^1^ | 5 | 1.12(0.92-1.37) | 0.247 | random | 56.1 | 0.059 |
| high grade^2^ | 6 | 1.18(0.98-1.42) | 0.090 | random | 59.6 | 0.030 |
| ***Different patterns of baldness*** |  |  |  |  |  |  |
| Frontal vs. no baldness | 13 | 0.93, 0.76-1.13 | 0.453 | random | 58.2 | 0.004 |
| Vertex with/without Frontal vs.no baldness | 9 | 1.18 , 0.96- 1.46 | 0.322 | fixed | 30.7 | 0.173 |
| Vertex without Frontal vs. no baldness | 3 | 1.23, 0.95-1.60 | 0.124 | random | 58.1 | 0.092 |
| Frontal with Vertex vs. no baldness | 5 | 1.00, 0.88-1.14 | 0.997 | fixed | 2.6 | 0.392 |
| ***baldness assessment way*** |  |  |  |  |  |  |
| self-reported | 8 | 1.04, 0.84-1.29 | 0.735 | random | 67.9 | 0.058 |
| trained observers | 4 | 1.07, 0.82-1.41 | 0.610 | random | 74.1 | 0.055 |
